# THE CHALLENGES OF LARGE QUALITATIVE DATASETS AND MEETING THE EXPECTATIONS OF INDIGENOUS COMMUNITIES AND FUNDERS

**DOI:** 10.1093/geroni/igad104.0988

**Published:** 2023-12-21

**Authors:** Jordan Lewis

**Affiliations:** University of Minnesota Medical School, Duluth campus, Duluth, Minnesota, United States

## Abstract

Literature on Alaska Native Elders and how they subjectively define a successful older age is limited. The lack of a culturally specific definition often results in the use of a generic definition that portrays Alaska Native Elders as aging less successfully than their White or non-Native counterparts. However, there is very little understanding of the diverse array of successful aging experiences and how this data will translate to other racial and ethnic minority populations, as well as advance the field of successful aging. This decade long study research the concept of successful aging from an Alaska Native perspective, or what it means to age well in Alaska Native communities and the lessons Elders. Qualitative, in-depth, interviews have been conducted with 154 Elders representing 20 participating communities to explore the concept of successful aging and the role of their community in the aging process. This study highlights the four (6) elements of successful aging, or “Eldership:” emotional wellbeing, spirituality, community engagement, Native ways of life, generativity, and physical health. These elements serve as the foundation of how communities define who is an Elder and what is important when considering who has aged successfully or not. This presentation will discuss challenges of analyzing large datasets to inform future studies, the development of a theory and measure of successful aging, as well as how to balance the needs of Indigenous community partners and the funders.

